# A qualitative investigation of HIV treatment dispensing models and impacts on adherence among people living with HIV who use drugs

**DOI:** 10.1371/journal.pone.0246999

**Published:** 2021-02-26

**Authors:** Taylor Fleming, Alexandra B. Collins, Geoff Bardwell, Al Fowler, Jade Boyd, M. J. Milloy, Will Small, Ryan McNeil

**Affiliations:** 1 British Columbia Centre on Substance Use, Vancouver, BC, Canada; 2 Interdisciplinary Studies Graduate Program, University of British Columbia, Vancouver, BC, Canada; 3 Department of Epidemiology, School of Public Health, Brown University, Providence, Rhode Island, United States of America; 4 Department of Medicine, University of British Columbia, St. Paul’s Hospital, Vancouver, BC, Canada; 5 Department of Medicine, Yale School of Medicine, New Haven, Connecticut, United States of America; 6 Program in Addiction Medicine, Yale School of Medicine, New Haven, Connecticut, United States of America; University of Lincoln-UK, UNITED KINGDOM

## Abstract

Antiretroviral therapy (ART) dispensing is strongly associated with treatment adherence. Among illicit drug-using populations, whom experience greater structural barriers to adherence, directly administered antiretroviral therapy (DAAT) is often regarded as a stronger predictor of optimal adherence over self-administered medications. In Vancouver, Canada, people living with HIV (PLHIV) who use drugs and live in low-income housing are a critical population for treatment support. This group is typically able to access two key DAAT models, daily delivery and daily pickup, in addition to ART self-administration. This ethno-epidemiological qualitative study explores how key dispensing models impact ART adherence among PLHIV who use drugs living in low-income housing, and how this is framed by structural vulnerability. Semi-structured interviews lasting 30–45 minutes were conducted between February and May 2018 with 31 PLHIV who use drugs recruited from an ongoing prospective cohort of PLHIV who use drugs. Interviews were audio-recorded, transcribed verbatim, and analyzed using QSR International’s NVivo 12 software. Interviews focused on housing, drug use, and HIV management. Models that constrained agency were found to have negative impacts on adherence and quality of life. Treatment interruptions were framed by structural vulnerabilities (e.g., housing vulnerability) that impacted ability to maintain adherence under certain dispensing models, and led participants to consider other models. Participants using DAAT models which accounted for their structural vulnerabilities (e.g., mobility issues, housing instability), credited these models for their treatment adherence, but also acknowledged factors that constrained agency, and the negative impacts this could have on both adherence, and quality of life. Being able to integrate ART into an established routine is key to supporting ART adherence. ART models that account for the structural vulnerability of PLHIV who use drugs and live in low-income housing are necessary and housing-based supports could be critical, but the impacts of such models on agency must be considered to ensure optimal adherence.

## Introduction

Access to antiretroviral therapy (ART) is critical to achieving individual-, community-, and population-level decreases in HIV-related morbidity, mortality, and viral transmission [1–3]. Optimal engagement in ART (i.e., ≥95% adherence rate) is recommended to achieve undetectable viral loads among people living with HIV (PLHIV) and to prevent further transmission [4], and is a central component of a combination HIV prevention and treatment strategy known as Seek, Test, Treat, and Retain (STTR) [1].

Despite global efforts to scale up access and adherence to ART, and overall engagement in HIV primary care [5, 6], some key affected populations, particularly people who use illicit drugs (PWUD), experience high rates of suboptimal ART outcomes, including lower rates of viral suppression [3, 7], and treatment interruptions [8, 9]. For example, one recent study found that engaging in informal income generation (e.g., sex work, drug dealing) and having a history of incarceration, both common among PWUD, were associated with reduced ART engagement [10]. Previous research has described how social and structural mechanisms drive suboptimal ART adherence [8, 11, 12]. More specifically, housing vulnerability has been implicated as a driver of adverse ART outcomes and disproportionately impacts PLHIV who use drugs (e.g., heroin, crystal methamphetamine) [13–15]. While housing is regarded as a critical determinant of health among PLHIV who use drugs, this population experiences a disproportionate burden of housing vulnerability, and is largely reliant on subsidized and low-income housing [13, 16]. Thus, PLHIV who use drugs living in low-income housing are an important population for treatment support programs.

Globally, persistent gaps in HIV treatment patterns remain prevalent among unstably housed persons and PWUD in well-resourced areas [17], including those settings that have implemented STTR strategies [16, 18, 19]. To address clinician concerns regarding adherence to self-administered ART routines (e.g., forgetting medication, ongoing drug use) [20], directly administered antiretroviral therapy (DAAT), in which PLHIV are directly given their medications by a health care professional for either immediate or later consumption, is often recommended to improve rates of adherence and viral suppression among PLHIV who use drugs [21, 22]. Within DAAT, PLHIV may be observed taking medications intended for immediate ingestion, and dispensed medications for later self-administered ingestion. Previous epidemiological research involving PLHIV who use drugs has found DAAT models to be associated with increased viral suppression, greater adherence, and positive immunological outcomes, although the observed impact disappeared when DAAT was discontinued [7, 21, 22]. Past qualitative research has characterized the utility of DAAT in empowering access and adherence to ART among structurally vulnerable PLHIV who use drugs [8, 23, 24], and the adherence-related benefits of such highly ritualized medication routines [20]. When paired with participation in an opioid agonist therapy (e.g., methadone treatment), the benefits of DAAT have been found to be amplified [7, 21, 24], and the co-location of these services is thought to prompt PLHIV who use drugs to remember to take their medications, thus retaining them in care [25]. While evidence appears to support this highly structured treatment model, questions still remain regarding how experiences and impacts of HIV treatment are framed by the social and structural inequities of PLHIV who use drugs.

In Vancouver, Canada, ART is readily accessible, as it is provided at no cost pursuant to the goals of province-wide STTR programming [26]. Here, PLHIV who use drugs are typically able to access three main treatment models: self-administration, daily delivery, and daily pickup, the latter two of which are forms of DAAT. Within daily delivery, a health care professional hand-delivers a day’s worth of medications to PLHIV at their home at a specified time each day, while daily pickup involves PLHIV travelling to a specific pharmacy each day to be dispensed their medications. Further, Vancouver is host to many HIV-specific programs and health services designed to be responsive to the needs of drug-using populations [27–30]. However, barriers to accessing and adhering to HIV treatment persist in this setting, which can complicate efforts to implement STTR [31–33], even within HIV specific housing programs [30]. Indeed, the identification of barriers that persist in spite of access to universal health care and ongoing STTR efforts will be crucial to informing the implementation and optimization of treatment approaches, as well as complementary social and structural interventions to ensure optimal engagement with HIV treatment for drug-using populations [10, 33].

This ethno-epidemiological study explores how key dispensing models (i.e., self-administration, daily delivery, and daily pickup) impact ART adherence among PLHIV who use drugs living in low-income housing, and how these experiences are framed by structural vulnerability. The concept of structural vulnerability draws attention to the many ways that social suffering is reproduced through the interaction of structural forces (e.g. drug criminalization, surveillance) and socio-cultural forces (e.g. anti-drug stigma, racism) by focusing attention on socio-structural factors that limit agency and produce vulnerability to risk and harm [34, 35]. Understandings of structural vulnerability stress that intersecting oppressions and inequities produce differential adverse outcomes within the drug-using population. Thus, it is an appropriate lens through which to understand social and structural dimensions of HIV treatment among PLHIV who use drugs, particularly when employed in concert with qualitative research methods that have the potential to illustrate how HIV treatment experiences are framed by underlying social-structural mechanisms by foregrounding the lived experience of PLHIV who use drugs [8, 30]. Such understandings are critical to inform targeted interventions to optimize treatment outcomes in ways that are responsive to the experiences of PLHIV who use drugs, and the social-structural forces shaping their lives.

## Methods

### Study design and setting

This ethno-epidemiological study draws on semi-structured qualitative interviews conducted with participants of the AIDS Care Cohort to Evaluate exposure to Survival Services (ACCESS). Ethno-epidemiology draws on ethnographic and epidemiological methods to allow for better understanding of how contextual factors impact health and social harms—in this case, how key dispensing models impact HIV medication adherence among structurally vulnerable populations [34]. ACCESS is an open prospective cohort study of PLHIV who use drugs operating in Vancouver since 2005, and has been described in detail elsewhere [4]. ACCESS participants are recruited through self-referral, snowball sampling, and street outreach, and are eligible to participate in if they are 18 years or older, HIV seropositive, and used illicit drugs in the previous 30 days at baseline. ACCESS participants complete a standardized interviewer-administered questionnaire and provide blood samples at baseline and biannual follow-up visits. The questionnaire elicits information on items such as sociodemographic characteristics, drug use, housing status, and health care service engagement.

This study was conducted at a storefront research office located in Vancouver’s Downtown Eastside (DTES) neighbourhood. This is also the site of biannual follow-up visits for cohort participants. The DTES is a low-income inner-city neighbourhood, and is Canada’s largest street-based drug market, characterized by high rates of poverty, drug use, and housing vulnerability, as well as a disproportionate burden of HIV risk and seropositivity [36]. As a result, the DTES is home to many health and social services, including street-based HIV outreach programs and innovative harm reduction interventions (e.g., syringe distribution, supervised consumption sites). Further, a limited number of units of HIV-specific housing in downtown Vancouver is available to PLHIV [37]. Between February and May 2018, we conducted qualitative interviews with 31 cohort participants who had previously reported living in low-income housing in the DTES. Ethics approval was obtained from the Providence Health Care/University of British Columbia research ethics board.

### Participants and data collection

Participants were eligible for the present study if they had completed a follow-up visit with ACCESS in the previous year, and had reported living in the DTES. Exclusion criteria included having no fixed address (e.g., staying in shelters or outside). Participants were recruited through phone calls conducted by an ACCESS staff member, who described the study and invited them to participate in an interview. Recruitment continued until all eligible participants had been contacted and invited to participate in an interview a maximum of two times, rather than until data saturation had been reached, and this was used as the primary criterium for suspending data collection. Of the eligible participants, recruitment was targeted towards individuals who had reported ART adherence rates of 70% or lower in any of the last three ACCESS follow-up visits (i.e., these individuals were among the first invited to participate), and were living in low-income housing. Adherence rates are determined via questions surrounding ART adherence in the ACCESS follow-up visits. A random name generator was used to assign pseudonyms to participants.

The lead author (TF) and a Peer Research Assistant (i.e., a member of the DTES community who uses/used drugs and is trained in research activities) co-led semi-structured qualitative interviews with 31 PLHIV who use drugs between February and May 2018. TF is a woman and a research coordinator with graduate level training in qualitative research. While having no direct knowledge of TF, ACCESS participants have well-established relationships with our research program through their involvement in the longitudinal cohort study. Interviews took place in a private room at our DTES storefront research office. TF explained the study to participants and obtained written informed consent before the interview. No participants refused to participate, or dropped out at a later date. An interview guide was used to facilitate discussion, which centred on housing conditions, drug use patterns, HIV treatment and management, and service engagement. Interviews lasted 30–45 minutes, and participants received $30 cash honoraria as compensation for their time. Interviews were audio-recorded and transcribed verbatim. There was a considerable degree of concordance in interviews, suggesting further data collection would have yielded few new insights.

### Data analysis

Interview transcripts were imported into QSR International’s NVivo 12 qualitative data analysis software and analyzed using deductive and inductive approaches [38]. A coding framework informed by *a priori* categories was developed based on topics in the interview guide, and used to facilitate analysis. The research team met regularly to discuss emerging categories and revise the framework as needed, with TF, GB, RM, and a research coordinator assuming responsibility for coding transcripts. Reflective notes made after each interview were triangulated with emerging categories to support credibility and trustworthiness of findings [39]. Team members were randomly assigned to code transcripts, such that each transcript was coded independently by two team members (i.e., investigator triangulation). Coding was then compared in NVivo to ascertain inter-coder reliability, and any variations in coding were discussed and resolved by consensus amongst the research team to ensure codes were being reliably applied throughout the analysis process [38]. Final categories established were comprised of the three key HIV medication dispensing models (i.e., self-administration, daily delivery, and daily pickup) to best describe impacts of key HIV medication dispensing models. Data were then re-coded to support trustworthiness of the reported findings [39].

## Results

A total of 31 PLHIV who use drugs were interviewed. Participants ages ranged from 29 to 61, with an average age of 47.6 years. Of our participants, 15 self-identified as women, 14 as men, and 2 self-identified as transgender, Two Spirit, or non-binary. All but one of our participants were currently taking ART, and over half indicated that they use illicit drugs daily. Participant characteristics are reported in Table 1.

**Table 1:** Participant characteristics.

	Participants (n = 31)
**Age**	
*Mean*	47.6
*Range*	29–61
**Gender**	
*Men*	14 (45%)
*Women*	15 (48%)
*Transgender*, *Two-Spirit*, *or non-binary*	2 (6%)
**Ethnicity**	
*White*	13 (42%)
*Indigenous*	16 (52%)
*Other*	2 (6%)
**Receiving ART**	
*Yes*	30 (97%)
*No*	1 (3%)
**Drug of choice**	
*Heroin*	11 (35%)
*Crack cocaine*	3 (10%)
*Crystal methamphetamine*	9 (29%)
*Other (e*.*g*., *cannabis*, *powder cocaine)*	8 (26%)
**Frequency of drug use**	
*Daily*	16 (52%)
*3–4 times per week*	7 (23%)
*One or fewer times per week*	8 (26%)
**Current ART model**^**a**^	
*Self-administration*	16 (52%)
*Daily delivery*	7 (23%)
*Daily pickup*	8 (26%)
**Receiving methadone maintenance therapy**	
*Yes*	14 (45%)
*No*	17 (55%)
**Social assistance as primary income source**	
*Yes*	30 (97%)
*No*	1 (3%)

^a^ Note participants may have previous experience with treatment models other than that which they currently reported using.

Results are organized around key dispensing models to better unpack experiences of HIV treatment, and how these models impact and are impacted by experiences of structural vulnerability. While themes such as agency, vulnerability, control, and power emerged, these are discussed across key dispensing models, and organized to maximize relevance and impact.

### Self-administration

Participants who self-administered ART (n = 16) stored their medications at home, and were responsible for self-administering them daily. Successful self-administration required a certain degree of stability and independence; therefore, participants who achieved optimal adherence (i.e., ≥95%) with this model were likely to be less structurally vulnerable (e.g., stably housed, connected to services) and to have been living with HIV long enough to have established effective routines to support adherence. Integrating ART into an established “*healthy*” routine was key to supporting adherence among participants. For example, ‘Terry,’ a 61-year-old white, man had been living with HIV for more than 30 years, and in that time, had entirely integrated ART into his daily routine:

*I live* [stay] *at home a lot and I do go out for breakfast at* [an integrated HIV care facility], *and lunch*, *and after that I go do my grocery or pick up my medication if I could remember it*. *And basically very normal*.

Participants described being more motivated and better able to establish treatment routines if they experienced minimal side effects and their medications were easy to take and tolerate (i.e., simplified regimens and smaller pills). More than other treatment dispensing models, participants emphasized that, under self-administration, adhering to medications was a conscious choice that they had to continue re-making daily. This was the case for ‘Louise,’ a 48-year-old Indigenous woman, who framed her ART adherence within the context of her overall health:

*When I chose to take care of my health over anything else is when I actually realized that my HIV medication is really important to me*. *And even if I did miss a dose I notice I feel it like I realize that it does make a big difference when you do miss a day*. *Having housing*, *a roof over my head*, *is the most important thing…Usually I just feel really sluggish and just kind of not sick but I just don’t feel right either* [if I miss a day], *something is not right … Usually it’s because I’ve slept in or I was out partying* [using drugs] *when I shouldn’t have been*.

For Louise and others, without commitment or supporting strategies (e.g., reminders from family, phone alarms), it was too easy to become non-adherent. Further, Louise’s narrative underscores the level of stability needed to effectively and confidently self-administer ART. For example, Louise had been living in a supportive housing building for the previous six years, and, as such, had the space to be able to develop a robust, yet flexible treatment routine. She contrasted this with previous experiences of housing instability, during which the necessity of prioritizing survival needs (e.g., food, avoiding dopesickness) over managing medications impacted her adherence.

ART self-administration offered participants the flexibility to structure their days as desired, although remembering to take medications was identified as a challenge to adherence. The demands of daily life (e.g. rushed schedules, interpersonal issues, caregiving responsibilities), as well as meeting daily survival needs (e.g., attending food programs, avoiding dope-sickness) were common reasons for forgetting a daily dose. Although, under self-administered treatment routines participants had the freedom to take their ART at a later, or more convenient time of day, if and when they remembered. Still, participants noted challenges in maintaining treatment routines due to their structural vulnerability. For example, participants described increases in their drug use as a method to cope with poor housing conditions (e.g., staying awake from fear of pests) and what they characterized as “*unstable*” life circumstances (e.g., extreme poverty, housing vulnerability). These increases in drug use made it hard to keep up with treatment routines, and could lead to forgotten doses among those who bore responsibility for dispensing their own medications. This could have serious health consequences, as ‘Maria,’ a 42-year-old Indigenous woman, acknowledged her HIV viral load had become detectable after having achieved viral load suppression, because her drug use interfered with her ART adherence:

*I was drugging*. *I was drinking and drugging*. *It was my choice* [to go off ART]. *Because I didn’t want to have to go back to my house*, *because like that’s where my pills were in*. *If I go back to my house I had to leave the block* [the drug centre of the DTES]. *I had my friend*, *and all this stuff… When I went off my meds that time*, *my viral load went high*. *Like my CD4s went down*, *down*, *down*. *But my viral load went high*, *high*, *high*. *Like and I think it was in 300s or something*. *I can’t remember which*. *But I was like okay*, *as long as I don’t get past… as long as I don’t get to 500*. *Like that*, *that’s*, *you know*, *like in my mind*, *that’s–*, *that’s death country… that’s where I don’t want to go*. *It’s hard*.

Maria noted that she previously relied on her partner to reminder her to take ART, and since their separation she forgot to take her medications more frequently. Ultimately, while self-administration offered participants greater agency in their HIV care and flexibility in dosing and scheduling, the lack of clinical or peer oversight also increased the potential for forgetting or for voluntarily stopping ART when experiencing treatment fatigue or other structural barriers to adherence. As such, more participants using this model reported intermittent periods of non-adherence. This also led some participants to consider other treatment mechanisms involving observed dosing (detailed below) that were more responsive to their needs.

### Daily delivery

Participants using the daily delivery treatment model (n = 7) received an ART medication delivery from their pharmacy or HIV-specific care program to their housing daily, which for many was co-dispensed with daily methadone maintenance therapy (MMT). While take-home dosing of methadone may be authorized in some circumstances, PWUD must be proven to be “socially stable” at the discretion of a physician and abstinent from any illicit drug use [40]; criteria which did not apply to our participants. Thus, participants receiving daily MMT deliveries were required to take their methadone under direct pharmacist supervision, and would usually take their ART medications at the same time, leading to reports of optimal adherence. However, changes made in 2014 to the provincial methadone program, which also included a replacement to the methadone formulation (from compounded methadone to a pre-made formula Methadose®) eliminated this option for some participants. Under the new program, MMT delivery could only be authorized for people with “severe mobility restrictions” [41]. This had implications for both ART adherence and MMT outcomes for ‘Eileen,’ a 54-year-old white woman who transitioned to a self-administered ART routine when the 2014 provincial changes made her ineligible for MMT delivery:

*My pharmacist* [used to] *bring it* [ART] *to me*, *make sure I take them*. *I was getting my methadone then*, *back then you can have your methadone*. *It was easy*. *Now you can’t no more because of that new Methadose®*. *The Methadose® is not strong and it doesn’t last as long… He* [pharmacist] *used to come to my place to give it to me*. *I’d rather they come to me*. *Now you have to have a special something*, *like you cannot walk*, *or you’re very sick*, *so the doctor has to put that on your prescription*. *It used to be easy right*?

Eileen now also had to contend with change intolerance to the Methadose® formulation, which she had to then get from her pharmacy each day before it closed, and no longer had the pharmacist oversight she preferred in managing her ART.

For those participants experiencing the temporary or permanent mobility impairments necessary to receive daily MMT deliveries with ART co-dispensing, this model accounted for barriers to adherence (e.g., mobility, public transit access, drug use) to promote optimal and long-term treatment adherence. ‘Lynda,’ a 39-year-old white woman had been receiving daily ART deliveries for seven years and reported never missing her treatment when it was delivered. She believed that “*if there ever is a time in my life where I don’t have the pharmacy coming to my door all the time then I’m worried about how I would take my medicine and how well I would do it*.” Further, for participants managing multiple concurrent health conditions, daily deliveries ensured access to regular health care and adherence to other medications.

However, the usefulness of this service was a function of when and where pharmacies would deliver ART medications. While Lynda maintained optimal ART adherence when receiving deliveries, her treatment was interrupted when, as a result of poor building conditions, she was briefly moved to an emergency shelter during renovations to her housing. Her pharmacy was unable to deliver to the shelter, and as a result Lynda was unable to take her medication until she returned to her housing, suggesting those experiencing the highest degree of housing vulnerability would be unable to access this treatment model. Other participants missed doses if they were not present in their room when the pharmacists arrived. Citing unhelpful staff members, participants expressed frustration when they were not told their medication had arrived when using common spaces or visiting other tenants. This could lead to interruptions in optimal ART adherence if participants continually missed deliveries. For example, ‘Nancy,’ a 49-year-old Indigenous woman, was not receiving her deliveries as scheduled. Nancy’s ART was co-dispensed with her MMT, and after missing four doses she was in such pain she decided “*what’s the fucking difference*?” and discontinued treatment for a while. She reasonably believed she should not be expected to wait around in her room for her ART while feeling so sick from missed MMT doses. Similarly, participant reports of having to wait for deliveries reinforced structural vulnerability by limiting ability to attend to other daily survival needs (e.g., income generation, access to health and social services). Thus, while this model addressed some common challenges to adherence (e.g., forgetting doses, taking medication on time), it also erected its own unique barriers to optimal ART adherence, and rendered some participants more vulnerable to health and social harms.

### Daily pickup

Participants who picked up their medication daily from either a pharmacy or an integrated HIV care facility (n = 8) expressed how important proximity to these services was to facilitating optimal ART adherence. Having a shorter distance to travel to pick up daily medications allowed participants to more easily integrate their ART into their daily routines, as they could “*get up*, *go get meds*, *go start your day*” with minimal impact on their daily activities (e.g., income generation, drug use). Some participants reported that physically going to the pharmacy reinforced ART as part of their daily routine more strongly than self-administration, and made optimal ART adherence more attainable. In fact, for some participants this routine was so ingrained that they reported needing *“to take my* [ART] *regimen so I can get on with my day*.” Often daily ART dispensation was voluntary. Similar to daily delivery models, these participants acknowledged that their ART adherence was higher and they were less likely to forget to take their medication when someone assisted in managing their treatment routine, because “*that’s a load off my head having to remember to take them* [ART] *or whatever*.” Further, participants considered the structure and pharmacist oversight offered by this model to be a strength, particularly when compared with self-administration. ‘Liam,’ a 56-year-old Indigenous man, was one of several participants who transitioned to daily pickup after experiencing suboptimal ART adherence when self-managing medications,

*I go there* [pharmacy] *daily because a few years ago we were trying to get my HIV under control because I wasn’t taking them* [ART medications] *consistently*. *Like I’d take them*, *not take them*, *you know*, *back and forth*. *So*, *she* [doctor] *and I decided that maybe we’ll try this out and it’s been working pretty good now*.

Participants whose daily ART pickup was paired with another needed service, such as a food program or pharmacy-based methadone dispensation, reported rarely, if ever, missing a dose. Food programs in particular were discussed as facilitating not only treatment adherence, but also management of side effects and medication tolerance (e.g., taking ART with food to avoid nausea), in addition to addressing a key health concern (e.g., food security). Overall, models that were flexible enough to accommodate participants’ structural vulnerabilities and shifting daily needs were the most successful and most favoured. For example, ‘Steven,’ a 60-year-old Indigenous man, received his medication from an Indigenous-specific health service that would make home deliveries if he did not stop by for his daily ART: “*They bring it to me if I don’t go there*. *They have my permission to put it on the stove* [of Steven’s unit].”

Despite the general satisfaction towards this model, participants still reported significant barriers to adherence when receiving daily ART doses at a pharmacy. Participants with mobility and other accessibility challenges (e.g., access to public transit) often experienced great difficulty in getting to their pharmacy, which resulted in missed doses. This was particularly relevant for Steven, who used a wheelchair, but whose building elevator was often out-of-service. As with daily deliveries, this model required participants to structure their days around their ART, which not only limited agency, but underscored participants’ status as PLHIV and made their HIV status a central part of their daily lives. Further, similar to those who self-administered their medications, participants who picked up their ART expressed that their drug use could at times disrupt daily routines or sense of time, cause them to forget to go get their medication, or sleep through scheduled doses.

## Discussion

In summary, our findings demonstrate how experiences of different ART treatment models are framed by, and in some cases, perpetuate the structural vulnerabilities of PLHIV who use drugs and thus underscore existing barriers to achieving optimal ART adherence. Models that accounted for these structural vulnerabilities were viewed as most beneficial to supporting ART adherence, and facilitated successful integration of ART into daily routines. Our findings also highlight the health implications of changes to degree of structural vulnerability that individuals experience within the context of HIV treatment and drug use (e.g., changes to housing status). Further, our findings demonstrate how treatment experiences were shaped by participants’ perceptions of agency over treatment routines, pointing to the importance of considering impacts on agency when engaging PLHIV who use drugs in ART, and more broadly, the HIV care continuum as a whole.

This study highlights the importance of supporting ART routines that account for the structural vulnerabilities of PLHIV who use drugs (e.g., housing vulnerability, access to health services). Participants who were able to maintain optimal ART adherence were often able to do so because their treatment routines fit naturally within their daily routines [7], and accommodated, or at least did not increase, their degree of marginalization (e.g., offering delivery when a client was unable to pick up their medications). This is aligned with previous research demonstrating that health service delivery models must be flexible enough to accommodate the changing needs of PLHIV who use drugs (e.g., mobility restrictions) to support optimal health outcomes [20, 42–44], and in the context of our study, optimal ART adherence. However, while previous research [12, 20, 45, 46], has documented similar causes of sub-optimal adherence (e.g., forgetting medication, prioritizing drug use) as our study, the discourse has often lacked an analysis of how the broader structural vulnerability of PLHIV who use drugs frames these treatment experiences. In this regard, participant narratives emphasized that ART models were unable to fully address structural barriers to optimal ART adherence, and in fact reinforced marginalization [47]. Most prominently, participants reported missing HIV medications due to changes in illicit drug use (e.g., frequency, source), regardless of treatment model, and that these changes in their drug use patterns and HIV treatment were shaped by social-structural-level factors (e.g., housing vulnerability, lack of agency) that are known to impact ART outcomes [11, 13, 15, 48]. Moreover, when ART models attempted to address the adherence-related impacts of changes in drug use patterns by placing medication administration in control of someone else (i.e., DAAT models), this paradoxically reinforced structural vulnerability by limiting other survival-related activities (i.e., daily delivery) or creating further accessibility challenges (i.e., daily pickup). Given both the documented benefits of integrated and flexible ART models [27, 29] tailored to the needs of PLHIV [44], and the relative shortcomings of existing standalone models, our findings reveal the need for treatment reforms, such as relaxing restrictions on requirements for daily ART delivery, to provide the types of comprehensive support PLHIV who use drugs need in maintaining optimal adherence.

Further, our findings demonstrate how intermittent periods of non-adherence owing to the limitations of any one treatment model can lead PLHIV who use drugs to transition to other treatment mechanisms that are more responsive to their current needs. While a higher degree of autonomy in health care models is generally viewed more favourably than those requiring a higher level of support [49, 50], our study suggests that self-administered ART is not necessarily a common goal. Participants who had experienced sub-optimal adherence while self-managing their medications described exercising agency by switching to DAAT models that were better aligned with their treatment needs. Whereas previous research has described negative perspectives of clinician oversight [51, 52], in this regard, it was considered a strength of these treatment models. Conversely, PLHIV who use drugs may transition from DAAT models to self-administered ART as their circumstances allow for lower levels of support (e.g., secure long-term housing) or require a less rigid treatment structure. However, transitions between ART models, particularly when imposed rather than chosen, may cause further harms. Specifically, transitions to self-administered treatment for PLHIV who use drugs who are ineligible for daily MMT delivery may itself serve as a barrier to optimal ART adherence by removing structure and clinical oversight of treatment routines, and can increase vulnerability by removing daily access to health services. Previous research has noted the dynamic nature of structural vulnerability among drug-using populations [53, 54], and how health service needs fluctuate in response to such changes [8]. Our findings build on this body of work to show how PLHIV who use drugs transition between ART models in response to changing treatment needs, and underscore the need for treatment mechanisms that are flexible enough to accommodate varying degrees of structural vulnerability without necessitating switches to an entirely new ART model.

Building upon previous research with PWUD on asserting agency in health care interactions [49, 51, 55, 56], our findings underscore how PLHIV who use drugs seek to negotiate agency within, and across, different ART treatment frameworks in a way that that is sensitive to their own treatment needs. For example, studies from both Holt [49] and McNeil et al. [54] show the relationship between user agency and capacity to comply with MMT regimens. Similarly, our participants expressed that ART mechanisms which constrained agency presented the greatest challenges in achieving optimal ART adherence. This was true regardless of which model participants adhered to, which suggests that medication self-administration may, counterintuitively, limit agency for some if they feel they do not have adequate control over their treatment regimens, despite seemingly being the treatment model in which users have the highest level of control. Thus, for PLHIV who use drugs requiring higher levels of support, opting in to a more structured treatment model (i.e., DAAT) may be viewed as asserting agency over health care; although such models have been shown to constrain agency and impact ART adherence in other ways (e.g., rigid scheduling) [12]. Ultimately, our findings demonstrate that PLHIV who use drugs are sensitive to their own health care needs [49], and willingly transitioning to an ART model that supports optimal adherence, either through providing a higher or lower level of support, is an example of exercising agency in health care. These findings highlight that when navigating ART models, impacts on agency must be considered to ensure optimal adherence among PLHIV who use drugs, and HIV care providers should work with PLHIV who use drugs to ensure agency is preserved across HIV treatment.

### Study limitations

This study has several limitations. Given that this work recruits from a longstanding cohort study, it is likely that our sample population is over-representative of PLHIV who are well-connected to healthcare and other services relative to the general population of PLHIV who use drugs in Vancouver. This recruitment method may disproportionally exclude people who are unstably housed, not engaged in care, or not accessing other health and social services [57]. Therefore, our findings may not be transferable PLHIV who use drugs experiencing among the highest degree of structural vulnerability. Further, transgender, non-binary, and Two-Spirit participants, who are disproportionately impacted by HIV [58], were underrepresented in our study. Thus, findings may not reflect their specific experiences of ART treatment models. Lastly, while Indigenous people were over-represented, their experiences of ART treatment models did not directly speak to their lived experience as Indigenous. Future research should examine how intersecting marginalized identities (e.g., transgender, non-binary, and Two-Spirit; racialized persons) impact experiences of key HIV treatment models.

## Conclusions

In conclusion, our findings indicate how ability to maintain optimal ART adherence under any treatment model (i.e., self-administration, daily delivery, daily pickup) vary in response to shifting structural vulnerabilities, often necessitating transition between models to support treatment routines. ART models that account for the structural vulnerability of PLHIV who use drugs and live in low-income housing are necessary, and housing-based supports could be critical, but the impacts of such models on agency must be considered to ensure optimal adherence.

## Supporting information

S1 Checklist COREQ checklist. (DOCX)
