# Supplementary material for: A qualitative investigation of HIV treatment dispensing models and impacts on adherence among people living with HIV who use drugs
Source: PLoS One. 2021 Feb 26;16(2):e0246999. doi: 10.1371/journal.pone.0246999 (PMC7909635; doi:10.1371/journal.pone.0246999)
Supplement: S1 Checklist — (DOCX) [file pone.0246999.s001.docx]

**COREQ Checklist**

| **No.** | **Item** | **Report** |
| --- | --- | --- |
| **Domain 1: Research team and reflexivity** |  |  |
| Personal characteristics |  |  |
| 1 | Interviewer/facilitator | Participants and data collection |
| 2 | Credentials | Title page |
| 3 | Occupation | Participants and data collection |
| 4 | Gender | Participants and data collection |
| 5 | Experience and training | Participants and data collection |
| Relationship with participants |  |  |
| 6 | Relationship established | Participants and data collection |
| 7 | Participant knowledge of the interviewer | Participants and data collection |
| 8 | Interviewer characteristics | Participants and data collection |
| **Domain 2: Study design** |  |  |
| Theoretical framework |  |  |
| 9 | Methodological orientation and theory | Study design and setting |
| Participant selection |  |  |
| 10 | Sampling | Participants and data collection |
| 11 | Method of approach | Participants and data collection |
| 12 | Sample size | Participants and data collection |
| 13 | Non-participation | Study design and setting |
| Setting |  |  |
| 14 | Setting of data collection | Study design and setting |
| 15 | Presence of non-participants | Participants and data collection |
| 16 | Description of sample | Table I |
| Data collection |  |  |
| 17 | Interview guide | Study design and setting |
| 18 | Repeat interviews | N/A |
| 19 | Audio/visual recording | Study design and setting |
| 20 | Field notes | N/A |
| 21 | Duration | Study design and setting |
| 22 | Data saturation | Study design and setting |
| 23 | Transcripts returned | N/A |
| **Domain 3: Analysis and findings** |  |  |
| Data analysis |  |  |
| 24 | Number of data coders | Data analysis |
| 25 | Description of coding tree | Data analysis |
| 26 | Derivation of themes | Data analysis |
| 27 | Software | Data analysis |
| 28 | Participant checking | N/A |
| Reporting |  |  |
| 29 | Quotations presented | Results |
| 30 | Data and findings consistent | Results |
| 31 | Clarity of major themes | Results |
| 32 | Clarity of minor themes | N/A |
